# Soil inoculation of *Trichoderma asperellum* M45a regulates rhizosphere microbes and triggers watermelon resistance to Fusarium wilt

**DOI:** 10.1186/s13568-020-01126-z

**Published:** 2020-10-23

**Authors:** Yi Zhang, Cheng Tian, Jiling Xiao, Lin Wei, Yun Tian, Zhihuai Liang

**Affiliations:** 1 College of Bioscience and Biotechnology, Hunan Agricultural University, Changsha, Hunan China; 2 Institute of Agricultural Biotechnology Research, Hunan Academy of Agricultural Sciences, Changsha, Hunan China; 3 Institute of Plant Protection, Hunan Academy of Agricultural Sciences, Changsha, Hunan China

**Keywords:** *Trichoderma asperellum* M45a, Fusarium wilt, Rhizosphere microorganism, Watermelon

## Abstract

Fusarium wilt (FW) caused by *Fusarium oxysporum* f. sp. *niveum* (FON) is a soil-borne disease that seriously limits watermelon production. In the present study, *Trichoderma asperellum* (*T. asperellum*) M45a was shown to be an effective biocontrol agent against FW. In a pot experiment, the application of 10^5^ cfu/g of *T. asperellum* M45a granules had an improved control effect on FW during the blooming period (up to 67.44%) in soils subjected to five years of continuous cropping with watermelon, while the average length of watermelon vines was also significantly improved (P < 0.05). Additionally, the acid phosphatase (ACP), cellulase (CL), catalase (CAT), and sucrase (SC) activities in the M45a-inoculation group were significantly higher than those in the control (CK) group, and transformation of the soil nutrients (total N, NO3-N, and available P) was significantly increased. Moreover, *T. asperellum* M45a inoculation reduced fungal diversity, increased bacterial diversity and especially enhanced the relative abundance of plant growth-promoting rhizobacteria (PGPR), such as *Trichoderma, Sphingomonas*, *Pseudomonas*, *Actinomadura*, and *Rhodanobacter*. Through functional prediction, the relative abundance of ectomycorrhiza, endophytes, animal pathotrophs, and saprotrophs in the fungal community was determined to be significantly lower than that observed in the M45a-treated soil. Correlation analysis revealed that *Sphingomonas*, *Pseudomonas*, and *Trichoderma* had the most differences in terms of microorganism abundance, and these differences were positively correlated with ACP, CL, CAT, and SC. These findings provide guidance for the use of fungicides to achieve microecological control of FW in continuously cropped watermelon plots.

## Introduction

Continuous cropping can result in stunted crop growth, disease aggravation, and decreased quality, and it can create a bottleneck that restricts the sustainable development of agriculture (Wu et al. 2009a, b). However, vegetable planting in facilities with single and continuous cropping is widespread in China due to the restriction of production and cultivation conditions and economic interests (Gao et al. 2019). Soil sterilization is the main measure to overcome the obstacles associated with continuous cropping production. Although this approach can alleviate obstacles, it causes substantial harm to the environment in the long run. Therefore, environmentally friendly methods to alleviate continuous cropping obstacles by regulating soil microbial communities are being explored (Umadevi et al. 2018).

Soil microorganisms and soil enzymes are important components of soil ecosystems and important indexes to evaluate soil ecological quality and soil fertility (Li et al. 2018). Moreover, continuous cropping often leads to changes in nutrient contents, soil enzyme activity, and other physical and chemical properties, as well as changes in the soil microbial environment, which restrict the absorption of nutrients in soil and can cause soil-borne diseases (An et al. 2011). The plant rhizosphere, which is the region of soil that adheres to plant roots, is the site of complex ecological and biological processes and is affected by root exudates (Fang et al. 2013; Philippot et al. 2013). Microorganisms in the plant rhizosphere play a key role in maintaining ecosystems, and the abundance and diversity of microbial communities are sensitive to fertilization, irrigation, and plant health (Mazzola, 2004; Raaijmakers et al. 2009; Zhao et al. 2018; Zhou et al. 2017). For watermelon, changes in the microbial rhizosphere community may affect the plant’s resistance to *Fusarium oxysporum* f. sp*. niveum* (FON) (García-Ruiz et al. 2008). In addition, soil enzyme activity plays an important role in nutrient cycling and reflects the total soil biological activity, which may be associated with plant diseases. Additionally, soil microorganisms are the promoters and participants of soil biochemical reactions and have a direct correlation with soil enzyme activities (Wang et al. 2018).

Watermelon (*Citrullus lanatus*) is one of the most popular summer fruit crops worldwide (Guo et al. 2013). However, watermelon growth and yields have been significantly reduced by several soil-borne diseases, such as Fusarium wilt (FW) (Zhang et al. 2005) and blight disease (Zhang et al. 2016). FW, which is one of the most harmful diseases, is caused by FON and leads to decreased yields (An et al. 2011). This fungal pathogen population can reside in the soil for a long time and be rapidly affect plant roots, which leads to necrosis and induces wilting during the later stages of infection, thereby reducing yields and limiting economic productivity (Liu et al. 2019).

*Trichoderma* species are opportunistic, act as biocontrol agents, have the ability to induce plant resistance, and promote plant growth; thus, some *Trichoderma* species has been used as effective biofertilizers and biocontrol agents for plants grown in greenhouses and fields (Hermosa et al. 2012; Pandey et al. 2016). For example, Yuan et al. (2018) demonstrated that five *Trichoderma* strains substantially facilitated the growth and improved the nutritional quality of orchard grass. Yang et al. (2011) also showed that *Trichoderma harzianum* (SQR-T307) is an efficient biological control agent against *Fusarium oxysporum*. He et al. (2019) found that *Trichoderma asperellum* GDFS1009 granules can promote growth and resistance to *Fusarium graminearum* in maize. In recent years, most studies on the microbial community of the soil rhizosphere have mainly focused on bacterial communities, and limited information is available on fungal communities (Yu et al. 2013; Zhao et al. 2014; Shen et al. 2015a, b; Shen et al. 2015a, b). However, a few studies have been conducted on the effect of *Trichoderma asperellum* on the growth of continuously cropped watermelon, particularly the microecological regulation mechanism in the rhizosphere during the pathogenesis of FW. The hypothesis is that the application of *Trichoderma asperellum* strain M45a (*T. asperellum* M45a) can change the compositions of the bacterial and fungal communities in the rhizosphere soil of continuously cropped watermelon. In this study, we used high-throughput sequencing to compare the rhizosphere microbial communities before and after treatment with *T. asperellum* M45a and explored the underlying mechanisms on plant growth promotion and disease (FW) suppression.

## Materials and methods

### Trichoderma strains and plant material

*Trichoderma asperellum* M45a (CCTCC NO: M2019513, China Center for Type Culture Collection) provided by the Hunan Agricultural Biotechnology Research Institute, Changsha, China, was used throughout our study. *T. asperellum* M45a was cultured in potato dextrose agar (PDA) broth at 28 °C. A *T. asperellum* M45a conidia suspension was prepared according to Zhang et al. (2013b), and the determined concentration was 7.5 × 10^6^ colony-forming units (cfu)/ml. The watermelon cultivar ‘Zaojia 8424′, which is susceptible to FON, was used to investigate FW disease incidence (Faheem et al. 2015) and the length of watermelon vines.

### Greenhouse experimental design and soil sample collection

In the greenhouse experiment, potting soil was collected from a field at the Hunan Academy of Agricultural Sciences in Hunan, China (lat. 28° 28′ 55″ N and long. 113° 20′ 58″ E), which had been subjected to five years of continuous cropping with watermelon. The clay soil contained 2.7% organic matter, 1818.68 mg/kg of total N, 438.13 mg/kg of available K, 73.26 mg/kg of available P, and pH = 4.6. The soil enzyme activity levels were as follows: 0.7 u/g ACP, 9.44 u·g^−1^ CL, 26.30 u/g CAT, 6.545 u/g SC, and 683.18 u/g UE. The concentration of FON in the soil was 5 × 10^3^ cfu/g, and the content of *Trichoderma* spp. was 1.77 × 10^4^ cfu/g. Two treatments were performed in the greenhouse experiment in May 2018: a *Trichoderma* treatment **(**200 ml of the *Trichoderma asperellum* M45a conidial suspension (7.5 × 10^6^ cfu/ml) was added to 15 kg of soil, and this mixture was sprayed and mixed to achieve a concentration of 1.0 × 10^5^ cfu/g in the test soil) and Control **(**add 200 ml of sterile water to 15 kg of soil**).** Each treatment included five plastic pots and 30 seeds per pot, and all the plastic pots were laid out randomly. All watermelon plants were grown in a greenhouse with temperatures ranging from 28 to 35 °C, with natural light during the day, and from 22 to 25 °C at night.

In addition, soil samples were collected from two treatments in June and July of 2018. Each rhizosphere soil sample was collected from five watermelon plants during the germination period (S1), the seedling period (S2), the smoke trailing period (S3), and the blooming period (S4). All the soil samples were sieved (2-mm mesh) and divided into two subsamples: one subsample was stored at 4 °C for soil enzyme activity and property analyses, and the other subsample was stored at − 60 °C for DNA extraction.

### Soil enzyme activities and properties

For each rhizosphere soil sample, the activities of the extracted soil enzymes were determined fluorometrically using methylumbelliferone (MUB)-linked model substrates with a TECAN Infinite M200 spectrophotometer (G10S UV–Vis, Thermo Fisher, USA), including soil acid phosphatase activities, soil urease (Emami et al. 2013), soil cellulase (Kizilkaya et al. 2012), soil sucrase, and soil catalase activities (Allison and Jastrow, 2006), which were detected at the following wavelengths: 450 nm for S-CAT and 365 nm for the other enzymes. Then, the contents of soil organic matter (OM) (method GB9834-1988), soil total nitrogen (TN), available nitrogen (AN) (modified Kjeidahl method, HJ/T 707-2014), soil available kalium (AK) (flame atomic absorption spectrophotometry method, GB 9836-1998), and soil available phosphorus (AP) (sodium hydrogen carbonate solution-Mo-Sb anti spectrophotometric method, HJ/T 704-2014) were measured by the Institute of Soil Science, Chinese Academy of Sciences (Nanjing, China).

### Quantitative PCR analysis of Trichoderma spp. and FON

The abundances of *Trichoderma* spp*.* and *F. oxysporum* f. sp. *niveum* (FON) in the rhizosphere soil and root tissue of the watermelon plants were estimated using real-time PCR assays with an IQ5 Real-time PCR system (Bio-Rad Lab, LA, United States). The *Trichoderma* species were quantified using quantitative PCR with DG/DT primers (5′-CTGGCATCGATGAAGAACG-3′/5′-ATGCGAGTGTGCAAACTACTG-3′) (Wei et al. 2013). The FON species was preamplified by PCR for 18 cycles by Fonq-F/Fonq-R(5′-GTTGCTTACGGTTCTAACTGTGC-3′/5′-GGTACTTGGAAGGAATTGTGGG-3′), and then fluorescence quantitative PCR was performed using 1 μl of PCR product as a template in our laboratory (Xiao et al. 2018). The initial copy number of the target gene was calculated by comparing the threshold cycle (Ct) values of each sample with the calibration curve. The *Trichoderma* standard equation was set up for is y = − 0.3328x + 12.551, R^2^ = 0.9865 (y: the threshold cycle; x: the log of the spore concentration), and the calibration curve for FON was fixed according to the methods suggested by Xiao et al. (2018). Sterile water was used as a negative control. All amplifications were conducted three times.

### Rhizosphere soil DNA extraction and sequencing

DNA from each rhizosphere soil sample was extracted from 100 mg of soil using a MoBio kit (MO BIO Laboratories, Inc., USA) according to the manufacturer's instructions. The quality of the DNA samples was measured using a NanoDrop spectrophotometer (2000/2000C, Thermo Scientific, USA), and 2 μL of each sample was subjected to electrophoresis on a 0.8% agarose gel using a 1 × TAE buffer. Three replicates for each treatment were obtained in our experiment.

For the bacterial community analysis, the bacterial 16S rRNA gene was amplified using the following primers: 338F (5′-ACTCCTACGGGAGGCAGCA-3′) and 806R (5′-GGACTAC HVGGGTWTCTAAT-3′) (Zhao et al. 2014). For the fungal community analysis, the internal transcribed spacer (ITS) regions were amplified using the following primers: ITS 5F (5′-GGA AGTAAAAGTCGTAACAAGG-3′) and ITS 1R (5′-GCTGCGTTCTTCATCGATG C-3′) (Lopez-Mondejar et al. 2010). The DNA library was prepared with the TruSeq Nano DNA LT Library Prep Kit for Illumina. Sequencing of the paired-end library was performed using the Illumina MiSeq PE250 sequencing platform. The raw sequence data have been submitted to the NCBI sequence Read Archive (SRA) under accession numbers SRP265681 and SRP265697.

### Data analysis and statistical analyses

Raw bacterial and fungal sequences were assigned to each sample based on the corresponding unique barcodes. The sequences were clustered into operational taxonomic units (OTUs) at 97% sequence similarity by utilizing UCLUST (Edgar 2010) after quality control and elimination of short reads, chimaeras, ambiguous sequences, and low-quality sequences using QIIME (Caporaso et al. 2010). The OTUs were classified using the UNITE and Greengenes databases for fungi and bacteria, respectively (Desantis et al. 2006; Urmas et al. 2013). To analyse the differences in the bacterial and fungal community structures among the different treatments, principal coordinate analysis (PCoA) (Jiang et al. 2013) and redundancy analysis (RDA) (Jongman et al. 1995) were applied. Differences in the parameters among treatments were compared by performing one-way analysis of variance (ANOVA) at the end of each bioassay followed by Duncan's multiple range tests in IBM SPSS Statistics 25.0 (P < 0.05). A value of P < 0.05 was regarded as significant.

## Results

### Effect of T. asperellum M45a on watermelon health properties and FON content

**Fig. 1:**
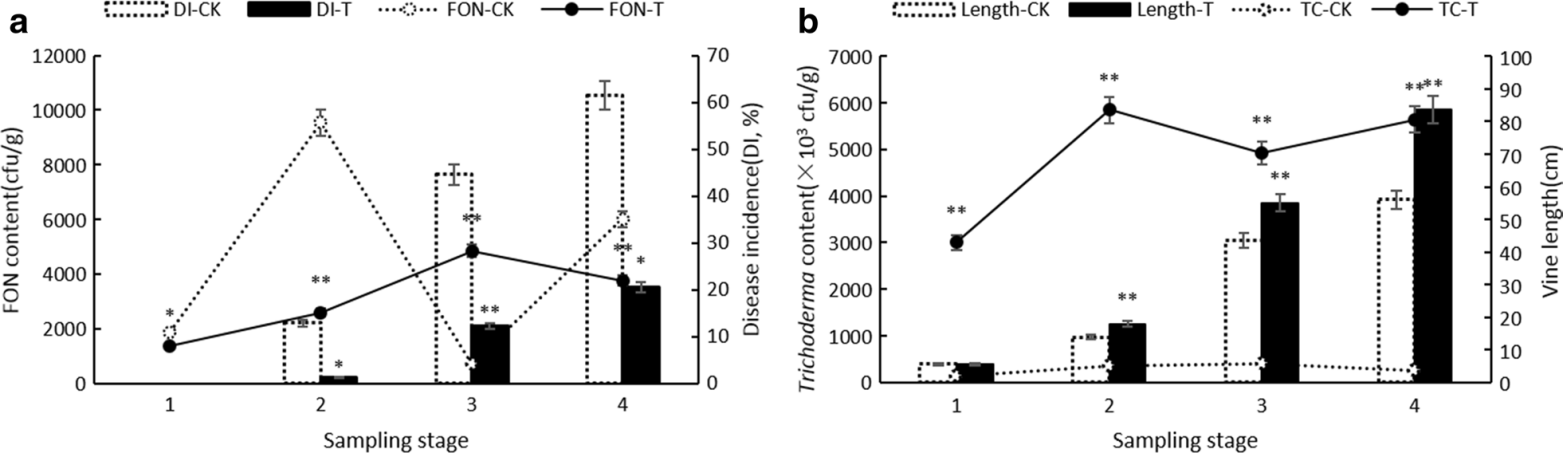
Effect of inoculating M45a on watermelon health properties and FON content. The inoculation of *T. asperellum* M45a in continuous cropping soil (T). The non-inoculated control (CK). S1: the germination period; S2: the seedling period; S3: the smoke trailing period; S4: the blooming period

The effect of *T. asperellum* M45a on watermelon health parameters was assessed based on the disease incidence (DI) of FW and the length of the watermelon vines. FW disease occurs in the seedling stage and then erupts rapidly until it becomes stable in the flowering stage. Compared to the CK treatment, the biocontrol effects of *T. asperellum* M45a on watermelon FW disease were 89.65%, 72.62%, and 66.72% during the seedling period (S2), the smoke trailing period (S3), and the blooming period (S4), respectively, and these results were significantly different from those of the CK group during the same periods (*P* < 0.01) (Fig. 1a). Additionally, the vine lengths of the watermelons inoculated with strain M45a also increased significantly by 29.44%, 26.43%, and 49.15% in the S2, S3, and S4 periods, respectively (Fig. 1b).

To understand the dynamic colonization relationship between *Trichoderma* spp*.* and FON in the rhizosphere soil, the qPCR technique was applied. We found that the number of FON in the CK treatment rapidly increased to 9543.66 cfu/g in the germination period (S1), which was significantly higher than of the *T. asperellum* M45a treatment. Except for the smoke trailing period (S2), the number of FON in the control rhizosphere soil was significantly higher than that in the M45a treatment (Fig. 1a). In addition, the contents of *Trichoderma* spp. in the rhizosphere soil of the M45a treatment were 22.07, 16.64, 12.35, and 23.05 times higher than those of the CK treatment during the same stage, respectively, and remained at 3.00 × 10^6^–5.64 × 10^6^ cfu/g for the onset of FW (Fig. 1b).

### Effect of T. asperellum M45a on soil enzyme activities and properties in rhizosphere soil

**Fig. 2:**
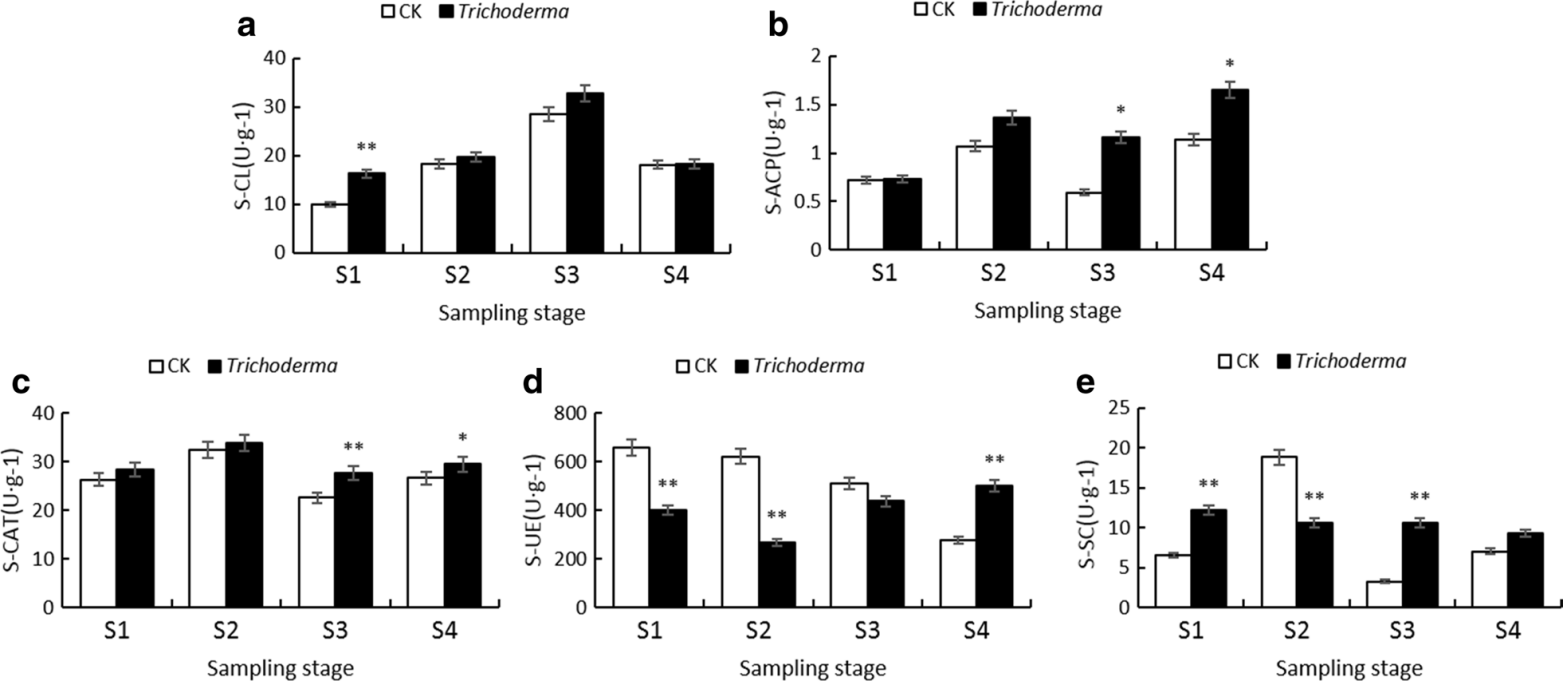
Effect of inoculating M45a on soil enzyme activities in the rhizosphere of watermelon. The inoculation of *T. asperellum* M45a in continuous cropping soil (*Trichoderma*). The non-inoculated control (CK). S1: the germination period; S2: the seedling period; S3: the smoke trailing period; S4: the blooming period

In the study, the enzyme activities of CL, ACP, CAT, and SC in the rhizosphere soil were increased following treatment with M45a, except for the activities of SC in the seedling period (S2) (Fig. 2). For example, the maximum increases in the activities of SC (224.15%) and ACP (95.80%) were obtained in the smoke trailing period (S3). Additionally, the activities of UE in the control group decreased gradually with the occurrence of watermelon FW. In contrast, the M45a treatment could effectively enhance the UE enzyme activity, and the highest activity (501.478 U/g) was observed in the S4 period. In addition, no significant difference was observed in the OM and available K, while the TN and available nutrients (NO3-N, and P) varied significantly in the different treatments (Table 1). Compared with the CK treatment, the M45a treatments significantly increased the contents of TN, NO3-N, and AP and decreased the AK content.

**Table 1:** Effect of inoculating M45a on soil enzyme activities in the rhizosphere of watermelon

Treatment	TC (%)	TN (mg/kg^−1^)	NH4^+^ (mg/kg^−1^)	NO3^−^ (mg/kg^−1^)	AP (mg/kg^−1^)	AK (mg/kg^−1^)
CK 1	2.72 ± 0.09a	2000.60 ± 6.86a	25.24 ± 0.60a	364.14 ± 15.50a	67.43 ± 1.99a	490.54 ± 9.46a
*Trichoderma* 1	2.65 ± 0.05a	2015.34 ± 3.40b	24.88 ± 1.12a	367.69 ± 4.67a	76.85 ± 0.77b	358.29 ± 6.64b
CK 2	2.21 ± 0.09a	1816.65 ± 4.10a	38.66 ± 0.51a	338.55 ± 11.20a	65.44 ± 1.87a	511.52 ± 4.64a
*Trichoderma* 2	2.33 ± 0.12a	2058.45 ± 5.76b	48.43 ± 2.01b	365.94 ± 6.55b	68.46 ± 1.35a	483.30 ± 8.31b
CK 3	2.51 ± 0.09a	1807.73 ± 7.46a	14.60 ± 1.13a	268.75 ± 8.21a	69.88 ± 1.53a	420.91 ± 5.63a
*Trichoderma* 3	2.27 ± 0.08ab	1920.03 ± 9.10b	30.38 ± 1.17b	283.62 ± 5.54ab	63.26 ± 2.18b	417.80 ± 5.93a
CK 4	2.74 ± 0.09a	1797.65 ± 13.09a	10.08 ± 0.80a	258.40 ± 10.04a	56.85 ± 0.88a	323.62 ± 17.40a
*Trichoderma* 4	2.58 ± 0.10a	1837.18 ± 6.58ab	9.38 ± 0.78a	287.23 ± 8.80ab	71.84 ± 2.21b	266.37 ± 6.75b

The application of *T. asperellum* M45a in continuous cropping soil (*Trichoderma*). The non-inoculated control (CK). *Trichoderma*1, *Trichoderma*2, *Trichoderma*3, *Trichoderma*4: the treatment with *T. asperellum* M45a at S1, S2, S3 and S4 period, respectively; CK1, CK2, CK3, CK4: the CK treatments at S1, S2, S3 and S4 period, respectively. S1: the germination period; S2: the seedling period; S3: the smoke trailing period; S4: the blooming period

^a^Suppression of M45a on FW disease and FON quantity in rhizosphere soil

^b^Promoting of M45a on watermelon growth and the change of colonization quantity in rhizosphere soil

### Effect of T. asperellum M45a on microbial community structure in rhizosphere soil

**Fig. 3:**
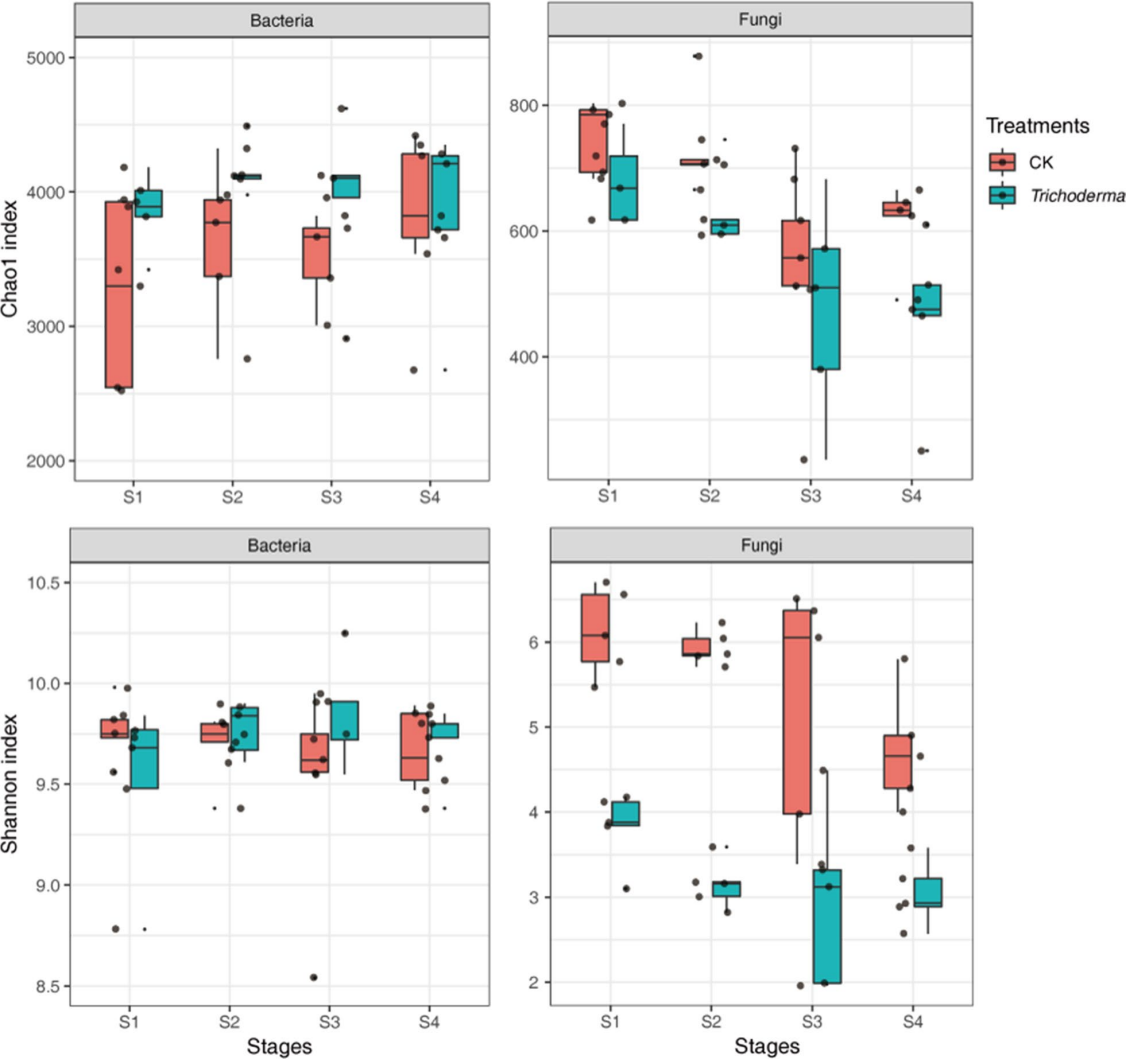
Richness (Chao1) and shannon diversity indexes for each treatment. The application of *T. asperellum* M45a in continuous cropping soil (*Trichoderma*). The non-inoculated control (CK). S1: the germination period; S2: the seedling period; S3: the smoke trailing period; S4: the blooming period

**Fig. 4:**
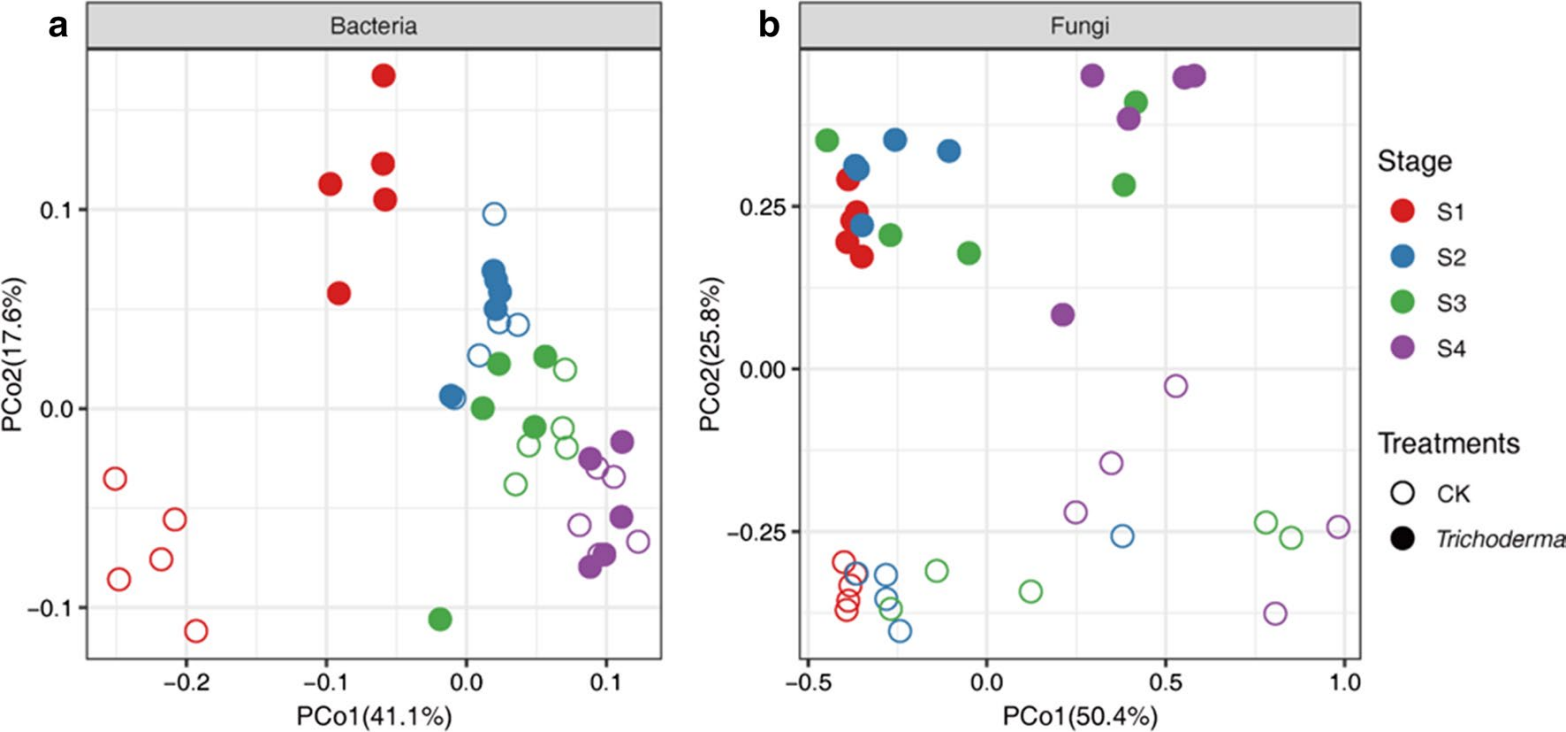
The PCoA result of the bacterial and fungal communities structure among the different treatment. The application of *T. asperellum* M45a in continuous cropping soil (*Trichoderma*). The non-inoculated control (CK). S1: the germination period; S2: the seedling period; S3: the smoke trailing period; S4: the blooming period

In total, 3,607,435 and 3,417,512 high-quality 16S and ITS sequences were obtained from the rhizosphere soil samples in the four stages, respectively. In the present study, the significant difference of alpha diversity between the two groups is shown in Fig. 3 and Additional file 1: Table S1. There was a significant difference in Chao1 between the group treated with M45a and the CK group (ANOVA, *p* < 0.05), and the fungal Shannon index values in the M45a group were significantly lower than those of the CK group at all stages (ANOVA, *p* < 0.01). With the occurrence of watermelon wilt, the fungal Chao1 values in the S1 and S2 groups were significantly higher than those of the S3 and S4 groups (Additional file 1: Table S1, *p* < 0.05). Additionally, the PCoA results showed that M45a(T) had a significant effect on the bacterial and fungal community compositions (Fig. 4). The first coordinate (PCoA1) showed differences of 41.1% and 50.4% in community variation, and PCoA2 explained 17.6% and 25.8% of the dissimilarity, respectively. In addition, these results were further verified by the results of PERANOVA dissimilarity tests based on the Bray–Curtis distance among the two groups (Bacterial: R = 0.05787, p = 0.001; Fungal: R = 0.27598, p = 0.001).

### Effect of T. asperellum M45a on the microbial community composition

**Fig. 5:**
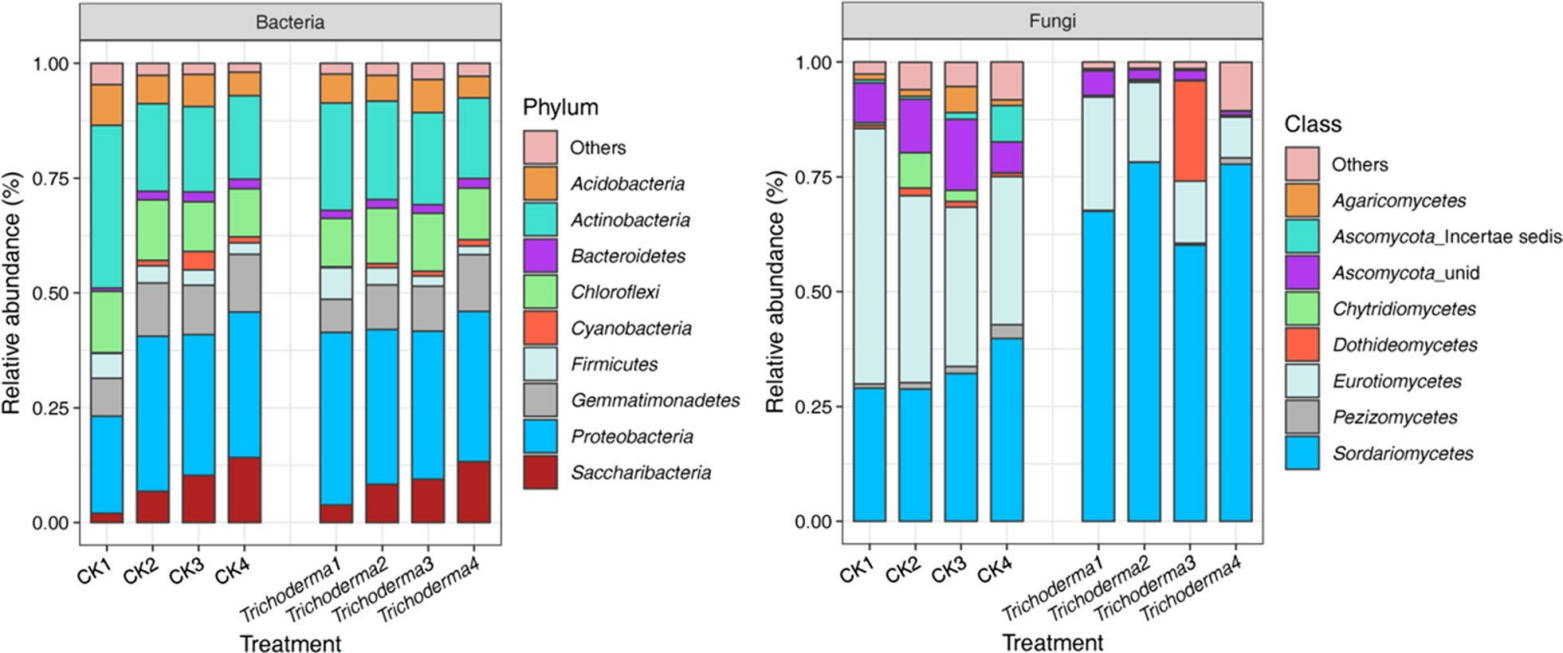
Relative abundances of bacterial phyla and fungal class under each treatment. The application of *T. asperellum* M45a in continuous cropping soil (*Trichoderma*). The non-inoculated control (CK). *Trichoderma*1, *Trichoderma*2, *Trichoderma*3, *Trichoderma*4: the treatment with *T. asperellum* M45a at S1, S2, S3 and S4 period, respectively; CK1, CK2, CK3, CK4: the CK treatments at S1, S2, S3 and S4 period, respectively

In general, the dominant bacterial phyla between two groups are visualized in the Fig. 5a and Additional file 2: Fig. S1. In brief, the most abundant bacterial phyla were *Proteobacteria*, *Actinobacteria*, *Chloroflexi*, *Gemmatimonadetes*, *Saccharibacteria*, and *Acidobacteria*, which contributed almost 86.7–91.8% of the bacterial sequences. While the rhizosphere communities were compared at different growth stages, *Saccharibacteria* (2.0–14.1%) was present at a significantly increased proportion in the rhizosphere with the onset of FW. At the genus level, among the top 20 genera, the relative abundance levels of *Pseudomonas*, *Sphingomonas, Actinomadura*, and *Rhodanobacter* in the M45a group were significantly higher than in the CK group (Additional file 2: Fig. S1, *p* < 0.05).

To investigate the difference in the fungal classes between the two groups, as illustrated in Fig. 5b, we found that the most relative abundant class was Sordariomycetes (60.12–78.67%) in the M45a group, which was significantly higher than that in the CK group. However, the second relative abundance of *Eurotiomycetes* was consistently lower in the M45a treatments (0.89–24.71%) than in the CK group (32.26–55.62%). For the fungal genera, the relative abundance levels of *Penicillium*, *Chaetomium*, *Aspergillus*, and *Acremonium* in the M45a-treated rhizosphere soil were significantly lower than those in the CK group (Fig. S2, *p* < 0.05). However, *Trichoderma* was present at a significantly increased proportion during the growth stages in the M45a treatment, consistent with the real-time PCR results.

### Effect of T. asperellum M45a on potential functional composition diversities

**Fig. 6:**
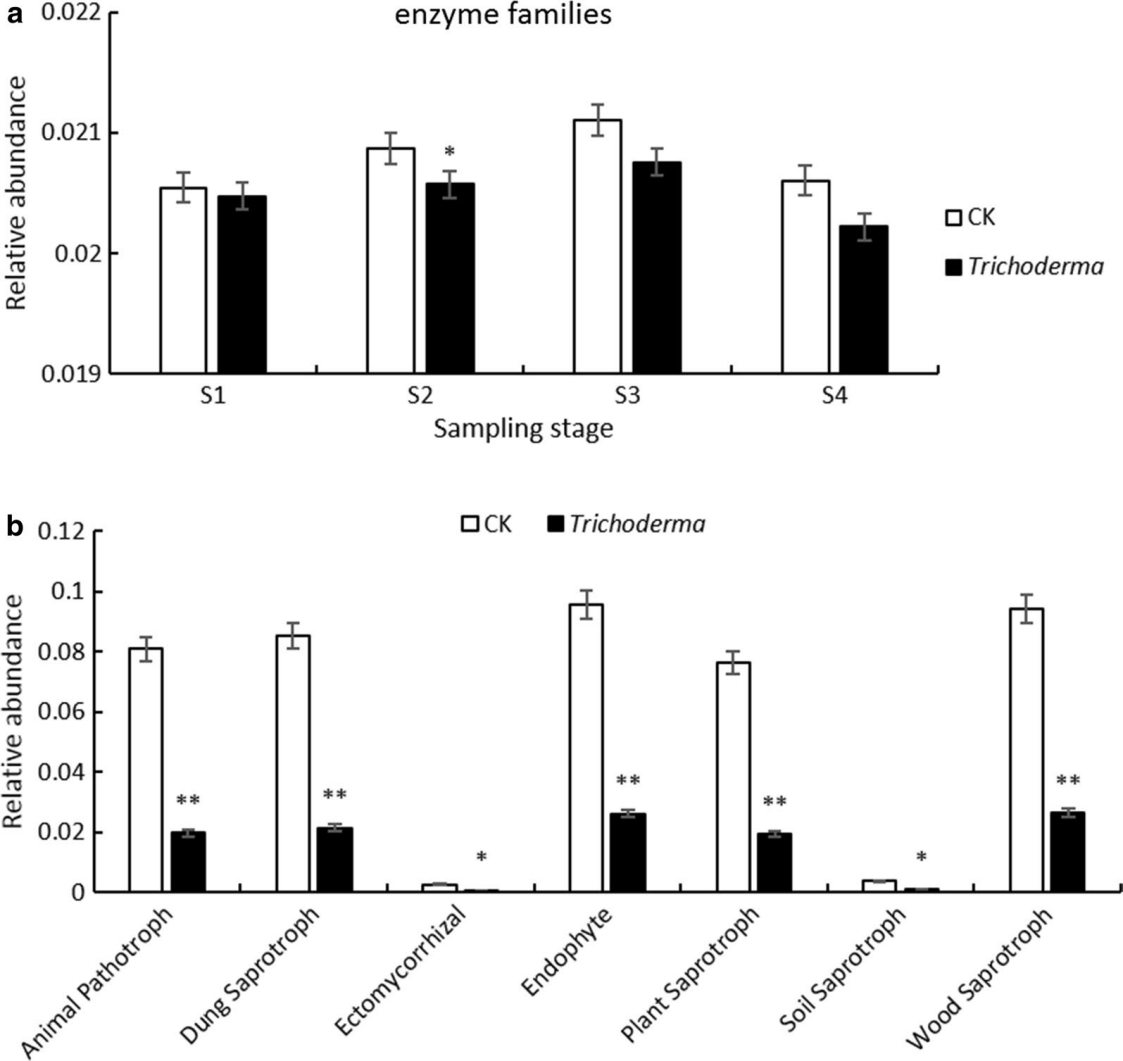
Relative abundances of bacterial metabolic pathways (**a**) and fungal trophic modes (**b**) identified from each treatment. The application of *T. asperellum* M45a in continuous cropping soil (*Trichoderma*). The non-inoculated control (CK). S1: the germination period; S2: the seedling period; S3: the smoke trailing period; S4: the blooming period

For the bacterial community, amino acid metabolism (10.81–10.98%), carbohydrate metabolism (10.45–10.81%), and energy metabolism (5.53–5.81%) were the main bacterial metabolic pathways in all treatments (Additional file 2: Fig. S3). In this study, the enzyme families (2.02–2.11%) were different between the M45a treatment and the CK group. Compared with the control soil, functional profiles with lower abundance were related to enzyme families in the M45a-treated soil during different stages, but there were no significant differences in response to disease (Fig. 6a).

For the fungal community, the different functional profiles of the trophic mode (symbiotroph, saprotroph, pathotroph) and guild (plant pathogen) of the fungal communities were compared for the M45a and CK soils during different stages. Compared with the CK soil, the relative abundance levels of ectomycorrhiza, endophytes, animal pathotrophs, and saprotrophs (dung saprotroph, plant saprotroph, soil saprotroph, and wood saprotroph) in the fungal community were significantly lower in M45a-treated soil (Fig. 6b). Additionally, the relative abundance levels of pathogens and other trophic modes showed no significant differences in these treatments.

### Relationships among soil enzyme activities, soil properties, and microbial communities

**Fig. 7:**
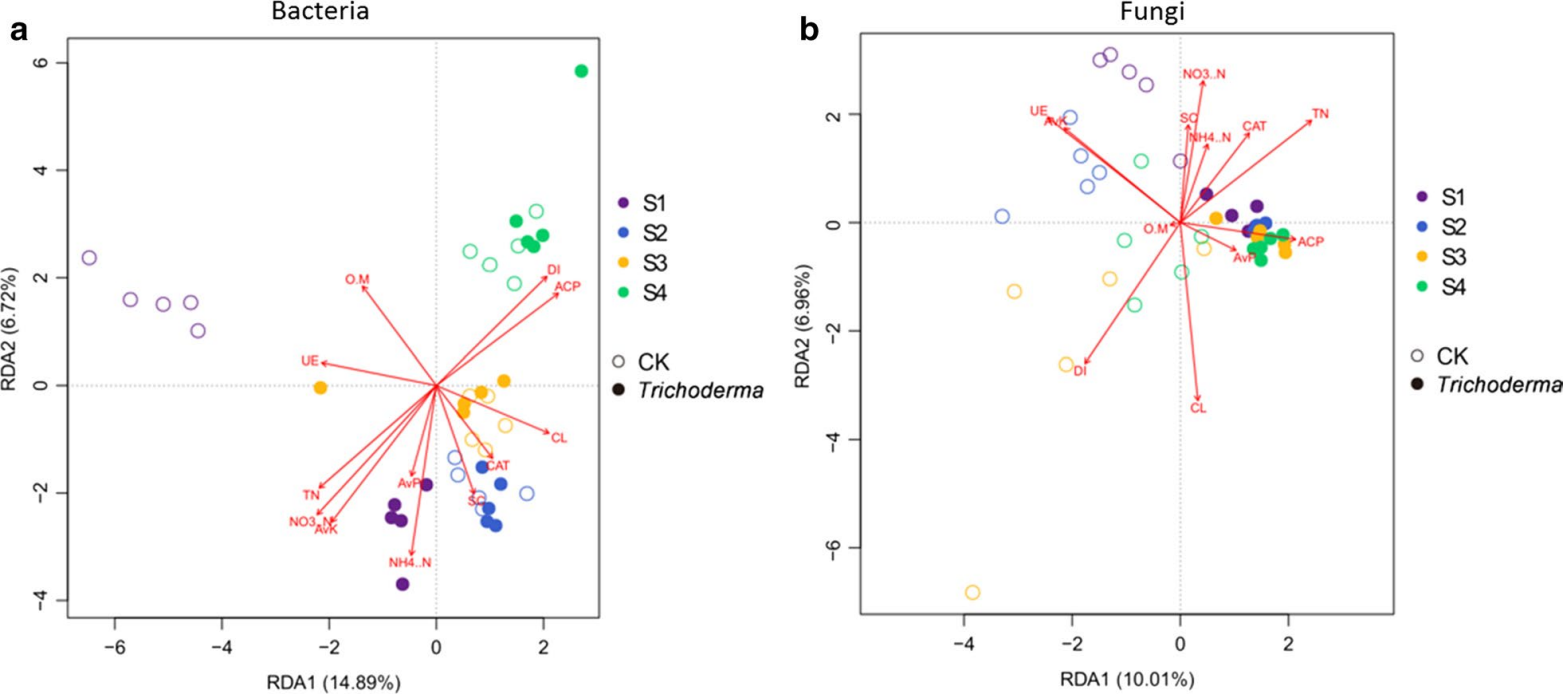
Redundancy analysis (RDA) of bacterial (**a**) and fungal (**b**) genera datasets and measured soil properties in rhizosphere soils after interactive forward selection (p < 0.05, VIFs > 20). The application of *T. asperellum* M45a in continuous cropping soil (*Trichoderma*). The non-inoculated control (CK). S1: the germination period; S2: the seedling period; S3: the smoke trailing period; S4: the blooming period

The RDA analysis showed that enzyme activities (CL, ACP, and CAT) and (AP) could greatly affect the microbial community composition in the rhizosphere soil (Fig. 7). In addition, a significant positive correlation was observed between the DI of FW and the CL activities. However, TN, NH4 + -N, and NO3-N were negatively correlated with the DI. Likewise, there were significant positive correlations between *Sphingomonas*, *Rhodanobacter*, *Pseudomonas*, *Gemmatimonsa*, and *Streptomyces* and the S-CL activities, and a significant negative correlation was found between *Nocardioides* and the S-CL activities (*p* < 0.05) (Additional file 1 Table S2). The correlations between the major fungal genera and the rhizosphere soil enzyme activities were then observed (Additional file 1: Table S3). Fungi are likely to be more sensitive to ACP activities than are bacteria; thus, the increased number *Trichoderma* spp. in the M45a treatment soil was sufficient to impose a stress on the fungi and to thereby influence fungi species such as *Penicillium*, *Chaetomium*, *Aspergillus*, and *Dendroclathra*, which were significantly negatively correlated with the ACP and SC activities in the rhizosphere soil, while the opposite trend was observed for the UE activities (*p* < 0.05).

## Discussion

Our results show that inoculation with *Trichoderma* significantly reduced the incidence of watermelon FW and increased the length of watermelon vines under continuous cropping. Many studies have reported that *Trichoderma* spp. exhibit significant effects against watermelon FW (Wu et al. 2009a, b), tomato late blight (Bahramisharif and Rose, 2018), rice sheath blight (Jambhulkar et al. 2018), maize stalk rot (Li et al. 2016), and banana wilt (Taribuka et al. 2017). Additionally, *Trichoderma* spp. have significant promoting effects on plants, seedlings and crop yields in some crops, such as those of cucumber, tomato and alfalfa (Zhang et al. 2013a, b; Liu et al. 2017; Zhang et al. 2019). *Trichoderma* spp. are adept at promoting plant growth, which is consistent with our result that the length of watermelon vines in the *T. asperellum* M45a treatment (*Trichoderma*) was significantly greater than that in the CK treatment. Several studies have demonstrated that *Trichoderma* spp. can increase the solubilization of soil nutrients (Yedidia et al. 2001; Harman et al. 2004). *Trichoderma* spp. can produce a large amount of organic acids, which release the delayed nitrogen and available nutrients (N, P, and K) in the soil for plant growth (Zhang et al. 2019). These findings explain our observation that the M45a treatment had greater contents of available N and P.

Enzyme activities have been proposed as biological indicators of soil quality and are closely related to soil function (Kandeler et al. 1996; Xian et al. 2015). The activities of CAT, CL, SC, UE, and ACP are closely related to the cycling of SOM, C, N, and P, respectively (Trasar-cepeda et al. 2007). In our study, M45a significantly increased the activities of CL and ACP, with significant differences observed in the spreading and flowering stages. Compared with the control group, the activities of CL, ACP, CAT, and SC in the soil treated with M45a were all increased, which indicated that *T. asperellum* M45a could promote the transformation of related enzyme activities such as N, C, and P in soil, thus promoting the decomposition of delayed nutrients in soil into available nutrients that can be absorbed and utilized by plants. These findings are consistent with previous studies and indicate that *Trichoderma* spp. could effectively increase the soil enzyme activity and improve plant growth (Zhang et al. 2018; Laur et al. 2018).

The control effect of *Trichoderma* spp. on watermelon FW was also reflected in the interaction between *Trichoderma* spp. and FON. After the application of *Trichoderma asperellum* to the field, the *Trichoderma* spp. spores can rapidly colonize and reproduce, further inhibiting the reproduction of FON (He et al. 2018). Thus, we further used real-time PCR to quantify the *Trichoderma* spp*.* and FON in the rhizosphere soil. The quantitative determination results show that the number of *Trichoderma* spp*.* in the rhizosphere soil treated with M45a was 10-fold higher than the control and remained stable during the entire process, while the amount of FON was greater than that in the control treatment only during the smoke trailing period (S3). The most likely reason for this difference is that a large number of FON could cause the occurrence of FW by infecting watermelon roots, while the M45a treatment can induce rapid proliferation of *Trichoderma* spp. around the rhizosphere of watermelon plants to effectively prevent FON infection in the roots.

As has been previously reported, soil microbial diversity is a key factor affecting soil quality and health (Zhao et al. 2018). Liu et al. (2019) found that continuous cropping led to increases in the diversity and richness of fungi in the rhizosphere soil. Therefore, balancing the rhizosphere soil microecology is crucial for healthy growth. Many studies have demonstrated that low fungal diversity and high bacterial diversity are observed after application of *Trichoderma* bio-organic fertilizer and Fen-Daqu (Zhang et al. 2016; Zhao et al. 2018). These results are consistent with the findings of our study, which showed that a significant difference was observed in the fungal diversity of the watermelon rhizosphere soil between the M45a and control treatments. The M45a treatment reduced the fungal diversity in the rhizosphere soil. To compare the different populations between the two groups at the genus level, we found that the relative abundance levels of *Penicillium*, *Chaetomium*, *Aspergillus*, and *Acremonium* in the M45a group were significantly lower than those in the CK group. We hypothesized that *T. asperellum* M45a could rapidly proliferate after inoculation, and reduce the relative abundance levels of *Penicillium*, *Chaetomium*, *Aspergillus*, and *Acremonium* through nutrient competition, thus regulating the structure and composition of the soil fungal community.

In addition, the soil bacterial and fungal community compositions varied in different treatments, and antagonism may occur between the inoculated *Trichoderma* and some bacteria (Pan and Jash, 2011). In our study, the abundances of *Sphingomonas*, *Pseudomonas*, *Actinomadura*, and *Rhodanobacter* increased significantly in the M45a treatment, which could potentially promote plant growth and health among the top 20 most abundant bacterial genera. Therefore, we hypothesized that the application of M45a may stimulate the proliferation of *Pseudomonas*, *Sphingomonas*, and *Rhodanobacter*, which are widely recognized as beneficial to plant growth and disease resistance (Cordier and Alabouvette, 2009; Laur et al. 2018; Carlson et al. 2019). Functional prediction showed that there was no significant difference in the bacterial metabolic pathways between the two treatments, which is similar to the results described by Benitez et al. (2017). However, fungi such as *Chaetomium* and *Acremonium*, which are saprotrophic or pathotrophic fungi that obtain nutrients by attacking host cells, were lacking in the M45a treated soil, so they may exert negative effects on other organisms (Nguyen et al. 2016). *Trichoderm* (Hu et al. 2016) and *Pseudomonas* (Wang et al. 2015) have been reported to efficiently control FW, so they may play a synthetic role in promoting plant resistance.

Soil properties have been considered to be an important factor for changing plant rhizosphere microorganisms in previous studies (Zhou et al. 2017). In this study, the RDA results show that M45a inoculation greatly influenced watermelon growth and the DI of FW in the pot experiment and could increase soil available N and P, which are directly beneficial to plant growth. These findings support our results that the soil available N and P contents were significantly higher in the M45a treatment than in the control treatment and that the M45a treatments significantly increased the length of the watermelon vines. The M45a treatment had a negative effect on the soil fungal community, which was most closely associated with changes in soil nutrients and enzyme activities. For example, *Trichoderma*, *Pseudomonas*, *Sphingomonas*, and *Rhodanobacter* were positively correlated with ACP, CL, CAT, and SC activities, which may indicate that soil enzyme activities are closely associated with living organisms (Govarthanan et al. 2018; Cruz et al. 2018). For species and functional analysis, metagenomic and transcriptomic techniques are required.

Therefore, M45a inoculation regulated the available soil nutrients, rhizosphere soil enzyme activities, and soil microbial community. Additionally, the inoculation indirectly improved crop growth and reduced the DI of FW in the continuously cropped watermelon. These results indicate that improving the plant rhizosphere microbiota can help control soil-borne diseases and promote plant growth. In future studies, we intend to study the microecological mechanisms in different soil environments by exploring the responses of *Trichoderma* to different soil environments. This will provide valuable information regarding the application of *Trichoderma* in different regions.

## Supplementary information

**Additional file 1: Figure S1.** The dominant bacterial genus differences among two different treatment. The application of *T. asperellum* M45a in continuous cropping soil (Trichoderma). The non-inoculated control (CK). Trichoderma1, Trichoderma2, Trichoderma3, Trichoderma4: the treatment with *T. asperellum* M45a at S1, S2, S3 and S4 period, respectively; CK1, CK2, CK3, CK4: the CK treatments at S1, S2, S3 and S4 period, respectively. S1: the germination period; S2: the seedling period; S3: the smoke trailing period; S4: the blooming period.** Figure S2.** The dominant fungal genus differences among two different treatment. The application of *T. asperellum* M45a in continuous cropping soil (Trichoderma). The non-inoculated control (CK). Trichoderma1, Trichoderma2, Trichoderma3, Trichoderma4: the treatment with *T. asperellum* M45a at S1, S2, S3 and S4 period, respectively; CK1, CK2, CK3, CK4: the CK treatments at S1, S2, S3 and S4 period, respectively. S1: the germination period; S2: the seedling period; S3: the smoke trailing period; S4: the blooming period. **Figure S3.** Relative abundances of bacterial metabolic pathways among two different treatment. The application of *T. asperellum* M45a in continuous cropping soil (Trichoderma). The non-inoculated control (CK).

**Additional file 2: Table S1.** The dissimilarity test of Richness (Chao1) and shannon diversity indexes for each period. The application of *T. asperellum* M45a in continuous cropping soil (Trichoderma). The non-inoculated control (CK). Trichoderma1, Trichoderma2, Trichoderma3, Trichoderma4: the treatment with *T. asperellum* M45a at S1, S2, S3 and S4 period, respectively; CK1, CK2, CK3, CK4: the CK treatments at S1, S2, S3 and S4 period, respectively. S1: the germination period; S2: the seedling period; S3: the smoke trailing period; S4: the blooming period. **Table S2.** Spearman correlation(r) coefficients between soil enzyme activities and dominant bacterial populations. * indicates that the significant value P < 0.05, ** indicates that the significant value P < 0.01. ACP: acid phospatase; CAT: catalase; CL: cellulase; UE: urease; SC: sucrase. **Table S3.** Spearman correlation(r) coefficients between soil enzyme activities and dominant fungal populations. * indicates that the significant value P < 0.05, ** indicates that the significant value P < 0.01. ACP: acid phospatase; CAT: catalase; CL: cellulase; UE: urease; SC: sucrase.

## Data Availability

The strains are available upon request. All data obtained have been included in the manuscript and the additional files.

## Abbreviations

FW: Fusarium wilt
DI: Disease incidence
ACP: Acid phospatase
CAT: Catalase
CL: Cellulase
UE: Urease
SC: Sucrase
OM: Organic matter
TN: Total nitrogen
AK: Available kalium
AP: Available phosphorus
PCoA: Principal coordinate analysis
ANOSIM: Analysis of similarities
