# Supplementary material for: Soil inoculation of Trichoderma asperellum M45a regulates rhizosphere microbes and triggers watermelon resistance to Fusarium wilt
Source: AMB Express. 2020 Oct 23;10:189. doi: 10.1186/s13568-020-01126-z (PMC7584699; doi:10.1186/s13568-020-01126-z)
Supplement: Supplementary file 1 — Additional file 1: Figure S1. The dominant bacterial genus differences among two different treatment. The application of T. asperellum M45a in continuous cropping soil (Trichoderma). The non-inoculated control (CK). Trichoderma1, Trichoderma2, Trichoderma3, Trichoderma4: the treatment with T. asperellum M45a at S1, S2, S3 and S4 period, respectively; CK1, CK2, CK3, CK4: the CK treatments at S1, S2, S3 and S4 period, respectively. S1: the germination period; S2: the seedling period; S3: the smoke trailing period; S4: the blooming period. Figure S2. The dominant fungal genus differences among two different treatment. The application of T. asperellum M45a in continuous cropping soil (Trichoderma). The non-inoculated control (CK). Trichoderma1, Trichoderma2, Trichoderma3, Trichoderma4: the treatment with T. asperellum M45a at S1, S2, S3 and S4 period, respectively; CK1, CK2, CK3, CK4: the CK treatments at S1, S2, S3 and S4 period, respectively. S1: the germination period; S2: the seedling period; S3: the smoke trailing period; S4: the blooming period. Figure S3. Relative abundances of bacterial metabolic pathways among two different treatment. The application of T. asperellum M45a in continuous cropping soil (Trichoderma). The non-inoculated control (CK). [file 13568_2020_1126_MOESM1_ESM.docx]

**Table S1 The dissimilarity test of Richness (Chao1) and shannon diversity indexes for each period.** The application of *T. asperellum* M45a in continuous cropping soil (*Trichoderma*). The non-inoculated control (CK). *Trichoderma*1, *Trichoderma*2, *Trichoderma*3, *Trichoderma*4: the treatment with *T. asperellum* M45a at S1, S2, S3 and S4 period, respectively; CK1, CK2, CK3, CK4: the CK treatments at S1, S2, S3 and S4 period, respectively. S1: [the germination period](javascript:;); S2: the seedling period; S3: the smoke trailing period; S4: the blooming period.

| **Period** | **Chao1** | **Shannon** | **Period** | **Chao1** | **Shannon** |
| --- | --- | --- | --- | --- | --- |
| **CK 1** | 744.102±51.931a | 6.100±0.454a | ***Trichoderma* 1** | 674.292±60.421a | 3.820±0.382a |
| **CK 2** | 721.964±74.936a | 5.990±0.153a | ***Trichoderma* 2** | 623.952±56.112a | 3.064±0.285a |
| **CK 3** | 570.332±85.3614b | 5.484±1.088a | ***Trichoderma* 3** | 469.058±152.274b | 2.890±0.9682b |
| **CK4** | 599.380±63.696b | 5.284±0.774a | ***Trichoderma* 4** | 454.054±116.532b | 2.870±0.397b |

**Table S2** **Spearman correlation(r) coefficients between soil enzyme activities and dominant bacterial populations.** * indicates that the signiﬁcant value P < 0.05, ** indicates that the signiﬁcant value P < 0.01. ACP: acid phospatase; CAT: catalase; CL: cellulase; UE: urease; SC: sucrase.

| **r** | **CAT** | **ACP** | **SC** | **UE** | **CL** |
| --- | --- | --- | --- | --- | --- |
| ***Sphingomonas*** | 0.2146 | 0.4220** | 0.3936* | -0.2852 | 0.2689* |
| ***Rhodanobacter*** | 0.2152 | 0.5732** | 0.5323** | -0.4163** | 0.3912* |
| ***Pseudomonas*** | 0.1272 | 0.2416 | 0.2756 | -0.3093 | 0.3455* |
| ***Gemmatimonas*** | -0.0985 | 0.4405** | 0.0501 | -0.2302 | 0.3263* |
| ***Streptomyces*** | 0.1343 | -0.3124* | -0.1552 | 0.1137 | -0.3754* |
| ***Jatrophihabitans*** | -0.2917 | -0.0649 | -0.3328* | -0.0338 | -0.2443 |
| ***Nocardioides*** | 0.1630 | -0.3332* | -0.3388* | 0.1 | -0.7602** |
| ***Dyella*** | 0.4302** | 0.0411 | 0.3268* | -0.4017* | 0.1503 |

**Table S3 Spearman correlation(r) coefficients between soil enzyme activities and dominant fungal populations.** * indicates that the signiﬁcant value P < 0.05, ** indicates that the signiﬁcant value P < 0.01. ACP: acid phospatase; CAT: catalase; CL: cellulase; UE: urease; SC: sucrase.

| **r** | **CAT** | **ACP** | **SC** | **UE** | **CL** |
| --- | --- | --- | --- | --- | --- |
| ***Trichoderma*** | 0.5293** | 0.3777* | 0.4844** | -0.5833 | 0.2608 |
| ***Penicillium*** | -0.0432 | -235233** | -0.4570** | 0.4081** | -0.5672** |
| ***Chaetomium*** | -0.3171* | -0.3587* | -0.5396** | 0.4060** | -0.3345* |
| ***Aspergillus*** | -0.2238 | -0.4430** | -0.3934* | 0.5159** | -0.2032 |
| ***Arthrographis*** | 0.2653 | 0.4411** | 0.4908** | -0.4387** | 0.2807 |
| ***Acremonium*** | -0.1092 | -0.5919 | -0.4786** | 0.4962** | -0.4261** |
| ***Talaromyces*** | 0.1049 | -0.4295** | -0.1555 | 0.4420** | -0.4694** |
| ***Dendroclathra*** | -0.2479 | -0.3799* | -0.4074** | 0.3123* | -0.2150 |
| ***Angulomyces*** | -0.0615 | -0.0393 | -0.1111 | 0.1201 | 0.3522* |
