# Supplementary material for: Soil inoculation of Trichoderma asperellum M45a regulates rhizosphere microbes and triggers watermelon resistance to Fusarium wilt
Source: AMB Express. 2020 Oct 23;10:189. doi: 10.1186/s13568-020-01126-z (PMC7584699; doi:10.1186/s13568-020-01126-z)
Supplement: Supplementary file 2 — Additional file 2: Table S1. The dissimilarity test of Richness (Chao1) and shannon diversity indexes for each period. The application of T. asperellum M45a in continuous cropping soil (Trichoderma). The non-inoculated control (CK). Trichoderma1, Trichoderma2, Trichoderma3, Trichoderma4: the treatment with T. asperellum M45a at S1, S2, S3 and S4 period, respectively; CK1, CK2, CK3, CK4: the CK treatments at S1, S2, S3 and S4 period, respectively. S1: the germination period; S2: the seedling period; S3: the smoke trailing period; S4: the blooming period. Table S2. Spearman correlation(r) coefficients between soil enzyme activities and dominant bacterial populations. * indicates that the significant value P < 0.05, ** indicates that the significant value P < 0.01. ACP: acid phospatase; CAT: catalase; CL: cellulase; UE: urease; SC: sucrase. Table S3. Spearman correlation(r) coefficients between soil enzyme activities and dominant fungal populations. * indicates that the significant value P < 0.05, ** indicates that the significant value P < 0.01. ACP: acid phospatase; CAT: catalase; CL: cellulase; UE: urease; SC: sucrase. [file 13568_2020_1126_MOESM2_ESM.docx]

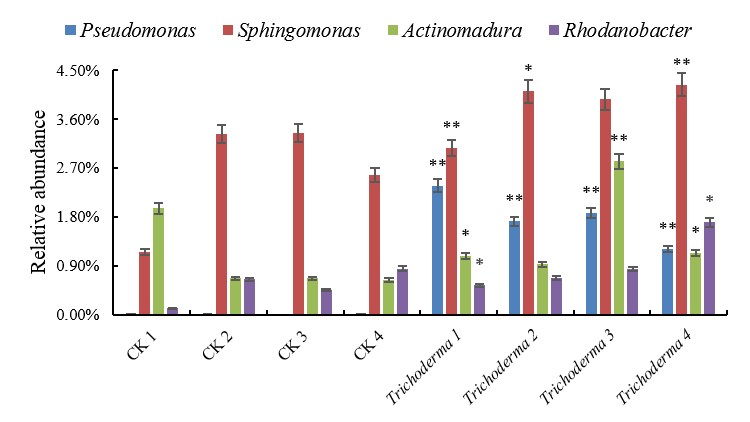


**Figure S1. The** **dominant bacterial genus differences among two different treatment.** The application of *T. asperellum* M45a in continuous cropping soil (*Trichoderma*). The non-inoculated control (CK). *Trichoderma*1, *Trichoderma*2, *Trichoderma*3, *Trichoderma*4: the treatment with *T. asperellum* M45a at S1, S2, S3 and S4 period, respectively; CK1, CK2, CK3, CK4: the CK treatments at S1, S2, S3 and S4 period, respectively. S1: [the germination period](javascript:;); S2: the seedling period; S3: the smoke trailing period; S4: the blooming period.


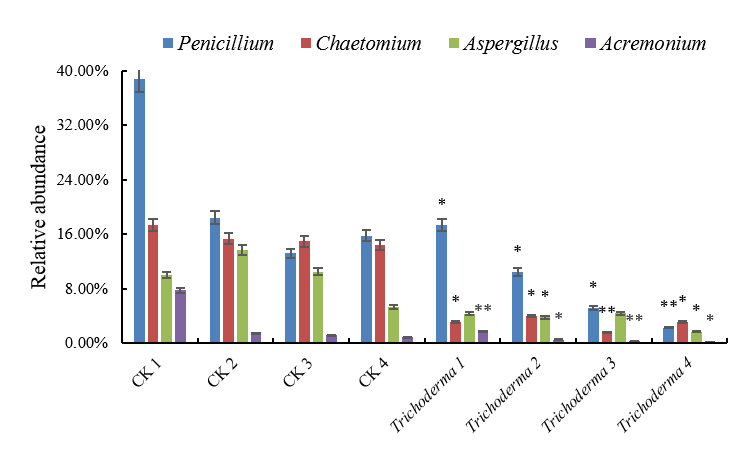


**Figure S2. The dominant fungal genus differences among two different treatment.** The application of *T. asperellum* M45a in continuous cropping soil (*Trichoderma*). The non-inoculated control (CK). *Trichoderma*1, *Trichoderma*2, *Trichoderma*3, *Trichoderma*4: the treatment with *T. asperellum* M45a at S1, S2, S3 and S4 period, respectively; CK1, CK2, CK3, CK4: the CK treatments at S1, S2, S3 and S4 period, respectively. S1: [the germination period](javascript:;); S2: the seedling period; S3: the smoke trailing period; S4: the blooming period.


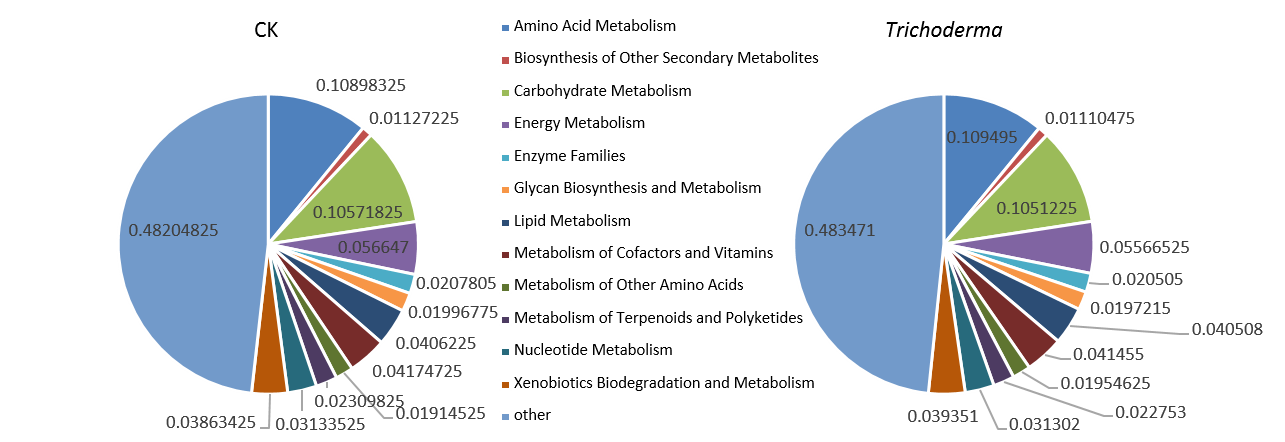


**Figure S3. Relative abundances of bacterial metabolic pathways among two different treatment.** The application of *T. asperellum* M45a in continuous cropping soil (*Trichoderma*). The non-inoculated control (CK).
